# The Factors Affecting Survival in Geriatric Hemodialysis Patients

**DOI:** 10.1155/2018/5769762

**Published:** 2018-07-10

**Authors:** Murat Tuğcu, Umut Kasapoğlu, Gülizar Şahin, Süheyla Apaydın

**Affiliations:** ^1^Department of Nephrology, Marmara University Pendik Training and Research Hospital, Istanbul, Turkey; ^2^Department of Nephrology, Ağrı Public Hospital, Ağrı, Turkey; ^3^Department of Nephrology, Sultan Abdulhamid Han Training and Research Hospital, Istanbul, Turkey; ^4^Department of Nephrology, Bakirkoy Sadi Konuk Training and Research Hospital, Istanbul, Turkey

## Abstract

**Introduction:**

The number of geriatric patients is increasing in hemodialysis population over the years and mortality is higher in this group of patients. This study evaluated the factors affecting geriatric hemodialysis patient survival.

**Materials and Methods:**

This retrospective cohort study enrolled patients discharged from our nephrology clinic from 2009 to 2014. Data collected included demographics, Eastern Cooperative Oncology Group-Performance Status, vascular access type, and metabolic parameters. Comorbidity was quantified using the modified Liu comorbidity index. The outcome measure was mortality.

**Results:**

The study enrolled 99 elderly dialysis patients (42.4% women (n = 42); mean age 75 ± 7 years). The mean follow-up duration was 19.7 ± 11 months. The mortality rate over the four years was 47.5% (n = 46). The modified Liu comorbidity index score, patient age, and Eastern Cooperative Oncology Group-Performance Status were significantly related to mortality in univariate and multivariate analyses.

**Conclusion:**

The present study revealed that comorbidities and low performance status at the onset of dialysis had shortened the survival time in the geriatric hemodialysis patient group.

## 1. Introduction

The elderly dialysis population has grown with the increase in the number of elderly people in many countries [1]. However, treatment decisions are difficult because the older dialysis population has a high burden of chronic health conditions and their limited life expectancy [2]. Several comorbidity index scoring systems have also been used to evaluate the prognosis of these patients objectively [3–6]. And, also, performance status generally reflects comorbidity burden and has prognostic significance, especially chronic diseases such as chronic kidney disease.

In fact, no established guidelines exist to inform the practice of hemodialysis in the elderly population. And also hemodialysis practice in elderly patients was different and tended to follow region-specific practices. This study is one of the few studies in this field in Turkey and evaluated the relationship between the Eastern Cooperative Oncology Group-Performance Status (ECOG-PS) and modified Liu comorbidity index (mLCI) score in survival in a geriatric hemodialysis population. We hypothesized that there would be negative correlations between comorbid diseases and performance status and survival outcome.

## 2. Materials and Methods

Geriatric dialysis patients (age > 65 years) who were started on hemodialysis between January 1, 2011, and December 31, 2014, at our nephrology clinic were analyzed retrospectively. Maintenance hemodialysis was defined as undergoing dialysis for more than 90 days and patients who died or switched from hemodialysis to peritoneal dialysis or transplanted were excluded. The hemodialysis is performed at the different private dialysis centers three times per week. The patients were followed from the first reported hemodialysis date to the date of death or December 31, 2014.

Patient demographic data, comorbidities, and date of death information were obtained from the National Dialysis Management System and computerized hospital records. Baseline metabolic parameters were taken at the 3rd month after starting of hemodialysis. Patient general health status before initiating dialysis was graded according to the ECOG-PS, ranging from 0 to 5, with 0 indicating that the patient is active and capable of normal everyday activity and 5 indicating that he or she is dead [7].

We calculated the mLCI (we allowed claims-based diagnosis capture to commence immediately upon initiating dialysis for a 90-day period). Briefly, this index assigns the following weights for 11 conditions: 1 point for atherosclerotic heart disease and diabetes; 2 points for cerebrovascular accident/transient ischemic attack, peripheral vascular disease, dysrhythmia, other cardiac diseases, chronic obstructive pulmonary disease, gastrointestinal bleeding, liver disease, and cancer; and 3 points for congestive heart failure (Table 1).

The data were analyzed using the Statistical Package for the Social Sciences for Windows 20.0 (SPSS, Chicago, IL, USA). Study subjects were censored if they were alive until December 31, 2014. The primary outcome (event) was death from any cause. Numeric data were presented as mean ± standard deviation. Either the Student's* t*-test or the Mann–Whitney U test was used for comparing the two groups. In univariate and multivariate analyses, mortality was the dependent variable and other variables were independent variables. We used the method of subtracting the mean to remove the multicollinearity produced by interaction of variables. Significance was set at* P *< 0.05.

## 3. Results

This study enrolled 99 elderly dialysis patients with a mean age of 75 ± 7 years; 42.4% were women (n = 42). The mean duration of follow-up was 19.7 ± 11 months. During the four-year study period, 47 (47.5%) patients died. Although most patients (68.7%) had chronic renal failure before initiating dialysis, a temporary hemodialysis catheter was the main vascular access (87.9%). Baseline metabolic parameters, primary renal disease, vascular access type, and gender did not differ significantly between survivors and nonsurvivors (Table 2).

Chronic comorbidities were very common in the nonsurvivors; the mean mLCI was 3, 7±2,3, and also the mean age (77.4±7.80 years) and ECOG-PS (2.8±1) were higher in this group. In the univariate and multivariate model, age, ECOG-PS, and mLCI score were significantly associated with mortality (Table 3).

**Figure 1:**
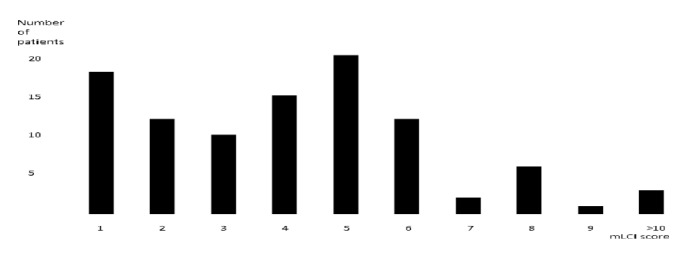
Distribution of mLCI score according to number of patients.

The mLCI score distribution is shown in Figure 1. Patients with low mLCI score had a significantly better survival rate than those with a high mLCI score (*p *= 0.027). Older age group were significantly associated with mortality (*p *= 0.047).

## 4. Discussion

Approximately one-third of elderly patients with end-stage renal disease (ESRD) have four or more chronic health conditions and most are not candidates for kidney transplantation [2]. When geriatric patients need dialysis, treatment decisions based on the patient's underlying condition and probable outcome are difficult [8, 9].

Mortality in elderly patients is closely correlated with many comorbidities independent of age [10, 11]. Therefore, multiple scoring systems have been developed and more than 80 randomized trials have been published to help physicians assess whether a dialysis patient could benefit from therapy and have their lifespan prolonged [12–15].

Liu et al. developed an improved comorbidity index for dialysis patients, the Liu comorbidity index (LCI) [16], which covers comorbid conditions, but not age, which is already a strong predictor of mortality. Recently, Rigler et al. modified the Liu comorbidity index because of the 270-day survival requirement for patient selection. Consequently, they could include sicker patients more prone to early mortality and reduce sample size loss without survivor bias. They found that the mLCI was as effective as the original [17].

Because mortality may be high in the first few months after initiating dialysis therapy, especially for elderly patients [1], we used mLCI for scoring our patients comorbidities and found that patients with higher mLCI scores had a shorter life expectancy (*p *= 0.009). Similar to our results, Khan et al. also found that patients in the highest LCI score group had the highest mortality risk and lowest survival rate [18].

Multiple comorbidities limit physical functioning; performance status is a vital instrument because it significantly influences morbidity and mortality [19]. Clinicians worldwide consider the ECOG-PS when planning new treatments for elderly patients [20]. We found that the ECOG-PS was related to mortality. Similar to our results, in a Japanese study, ECOG-PS was a prognostic factor in a multivariate analysis of hemodialysis patients aged ≥ 80 years [9]. However, we could not find a significant linear correlation between the mLCI score and ECOG-PS. However, the addition of functional status/fragility can help develop comorbidity scoring systems [21].

Age is a strong independent predictor in all existing scoring systems and we also found that older age was related to higher mortality. Another recent study found that dialysis may not benefit the survival of patients over 75 years old who have multiple comorbidities [22]. Nevertheless, older age should not be an obstacle to dialysis treatment because patients with a low mLCI may have an acceptable mean life expectancy.

This study has several important limitations. The number of subjects was small and we did not classify the severity of each comorbid condition. However, we recorded the ECOG-PS and it may indirectly quantify the severity of all comorbidities. And, also, the study was retrospective and the patients included were from a single institution.

## 5. Conclusion

Treatment of ESRD in geriatric population is complex and there is a lot of questions on how to best manage these patients. Our study evaluated the life expectancy of geriatric hemodialysis patients using mLCI score, age, and ECOG-PS. Our findings may help suggesting the prognosis of geriatric patients after starting dialysis and mortality is high in geriatric hemodialysis patients who have many comorbidities (i.e., higher mLCI scores), especially those with poor daily living status (i.e., higher ECOG-PS) and our findings contribute to international clinical experience.

## Figures and Tables

**Table 1:** Liu Comorbidity Index, 11 comorbid conditions, and weighing score.

***Comorbid Conditions***	***Weighing Score***
*Diabetic Mellitus*	1

*Coronary Artery Disease*	3

*Congestive Heart Disease*	1

*Cerebrovascular Disease*	2

*Peripheral Vascular Disease*	2

*Other Cardiac*	2

*Dysrhythmia*	2

*Chronic Obstructive Pulmonary Disease*	2

*Gastrointestinal Bleeding*	2

*Liver Disease*	2

*Cancer*	2

**Table 2:** Univariate analysis of clinical and metabolic parameters (at the 3rd month after starting hemodialysis). Significance was set at P < 0.05.

***Variables***	SURVIVORS N: 52	NON- SURVIVORS N: 47	***P***
***Age***	***73±6***	***77.4±7.80***	***0,012***

*Gender, Women (%)*	39	47	0,545

***mLCI score***	***2,5±2,2***	***3,7±2,3***	***0,009***

***ECOG-PS***	***2.3±0.8***	***2.8±1***	***0,023***

*Temporary Vascular Access (%)*	87	89	0,722

*Ischemic / Atherosclerotic (Primary Kidney Diseases)*	8	11	0,255

*Interstitial Nephritis (Acute / Chronic) (Primary Kidney Diseases)*	4	3	0,476

*Diabetic mellitus (Primary Kidney Diseases)*	16	12	0,149

*Unknown Etiology (Primary Kidney Diseases)*	10	17	0,24

*Other (Primary Kidney Diseases)*	8	8	0,65

*Serum Creatinine (mg/dl)*	7±3	6.1±2.4	0,11

*Serum Albumin (gr/dl)*	2.7±0.5	2.6±0.6	0,232

*Venous Bicarbonate (mmol/L)*	16.6±6	17.3±5.2	0,72

*Hemoglobin (g/dl)*	8.7±1.3	9.2±1.3	0,74

*C-Reactive Protein (mg/dl)*	5.1±5.7	6±7	0,77

*Ferritin (ng/ml)*	400±336	836±1855	0,10

*Proteinuria (g/day)*	3.2±3	2.6±2.5	0,36

*Cardiac Ejection Fraction (%)*	51	49	0,85

**Table 3:** Multivariate analysis of clinical and metabolic parameters. All variables in Table 2 were included in multivariate analysis, but only statistically significant results are presented in Table 3. Significance was set at *P* < 0.05.

**Dependent Variable**	**Multivariate Analysis**		
	Odds ratio	%95 CI	P value

Age	*5,520*	*1,8-18,1*	*0,021*

mLCI score	*4,944*	*1,6-12,2*	*0,029*

ECOG-PS	*3,986*	*2,09-5,15*	*0,049*
